# Biomineralization of coral sand by *Bacillus thuringiensis* isolated from a travertine cave

**DOI:** 10.1038/s41598-023-35893-z

**Published:** 2023-05-29

**Authors:** Yao Xiao, Huafeng Deng, Jianlin Li

**Affiliations:** Key Laboratory of Geological Hazards on Three Gorges Reservoir Area, Ministry of Education, College of Civil Engineering & Architecture, China Three Gorges University, Yichang, 443000 Hubei Province China

**Keywords:** Environmental microbiology, Civil engineering

## Abstract

Travertine is a typical product of microbial mineralization in the nature and its mineral composition is mainly calcite and aragonite. In this paper, *Bacillus thuringiensis*, a kind of mineralize bacterium is extracted from the travertine crystal to cenment coral sand, and the reinforcement effect of microbial induced carbonate precipitation (MICP) technology on coral sand under different cementation times is studied. Firstly, the culture conditions are optimized in nine pairs of trials, including urea content, microbial inoculation, shaker speed and incubation time. Under the optimal culture conditions, the coral sand is cemented by soaking method. With the increase of reinforcement times, the permeability coefficient of the sand sample is reduced to 10^−4^ cm/s, and the shear strength is increased by more than 130%. Compared with *Sporosarcina pasteurii*, the cohesion and internal friction angle of the coral sand column cemented by *Bacillus thuringiensis* are increased by more than 50% and 10%, respectively. The area distribution of T_2_ spectrum shows that with the increase of the number of cementation, the amplitude of the main peak decreases, indicating that the large pores are better filled, the number of medium and small pores are also reduced, and the pore area is significantly reduced, with the amplitude of about 44%. The above experiments verified that microorganism in travertine could also be used in MICP technology, and even achieve better reinforcement effect. It also provides a new way and idea for the selection of mineralized bacteria by MICP technology.

## Introduction

Engineering hazards such as dam foundation crack leakage^1^, heavy metal tailings pollution^2^, foundation liquefaction^3,4^ and slope instability^5^ often lead to property losses and even casualties. For a long time, relevant practitioners and scientific researchers have been committed to studying relevant measures to reduce the occurrence of such engineering problems. Biomineralization technology is a promising civil engineering technology developed on the basis of interdisciplinary research in recent years. It has good application prospects in dealing with such engineering problems. Microbial induced carbonate precipitation (MICP) is one of the typical representatives of biomineralization. This technology mainly utilizes some urea-hydrolytic microorganisms, which can form calcium carbonate with cementing function by driving urea hydrolysis and using carbonate ion and calcium ion in the solution. Under the action of MICP, the loose soil is cemented or the crack is sealed, so as to achieve the corresponding engineering requirements.

Currently, the main microbial species used in the MICP technology are *Bacillus pasteuris octadiae* (CGMCC 1.3687), *Sporosarcina pasterurii* (ATCC 11859), *Bacillus pasteuris octadiae* (DSMZ 33) and *Bacillus spheriformis* (LMG 22257). With the development in the study of soil reinforcement, many scholars independently separated different kinds of urea-hydrolytic bacteria and achieved a series of achievements in permeability reduction and strength improvement of soil. Chu et al.^6^ separated the *Bacillus sp.VS1* from tropical beach sand, and used the bacteria to strengthen large-sized sandy soils, the permeability resistance and mechanical properties of the reinforced soil foundation were improved. Qian et al.^7^ used the extracted *Bacillus S3* to cement the sand column, the recised compressive strength of the samples reached 1.9 MPa after the treatment, they utilized the separated *Bacillus S3* to reinforce the sandy soil, the compressive strength of the cemented sand approached 2 MPa. Khan et al.^8^ isolated the *Parahodobacter sp.* from the soil near beachrock and applied it to treat coral sand in the needle test, the estimated UCS of the specimens at some points could exceeded 7 MPa.

Carbonate minerals are widely distributed in the nature; for example, limestone, marble, dolomite. These types of rocks contain high amounts of calcium carbonate. In the nature, a variety of microorganisms can induce the formation of calcium carbonate during its metabolism. It's worth noting that travertine is one of the typical calcium carbonate products in this metabolic pathway. The carbonate precipitation is widely distributed all over the world^9^. In the movement process of geology and water system, the calcium ions in limestone stratum dissolve in groundwater constantly, making the groundwater holds a high content of mineral calcium. When groundwater overflows the earth's surface, the mineral calcium dissolved in the groundwater combines with H_2_O and CO_2_ in the environment to form calcium carbonate precipitation, and then convert to travertine. The study of microbe mineralization shows that microbe plays an important role in the formation of carbonate rocks. Fouke^10^, Sugihara et al.^11^ and Javad et al.^12^ found that the microbial widely exists in the travertine deposition environment, some kinds of extracellular metabolites produced by microorganisms during the growth process can capture and aggregate free Ca^2+^ or CaCO_3_ in water, and microorganisms serve as templates for nucleation and growth of calcium carbonate crystals^13^. In addition, microbial metabolism can promote the generation of travertine deposition^14^. Tugba et al.^15^ and Zhang et al.^16^ found that the formation of travertine can better seal the leakage passage, and can also re-bond broken or fractured carbonate stone structures in some rock formations to heal the fracture surface, so as to improve the impermeability and stability of underground structures.

At present, MICP technology has achieved great progress in the reinforcement of siliceous sand^17–20^, while the application of MICP technology in the treatment of coral sand^21^ has been less studied. Studies on the microstructure and shear resistance of the cemented coral sand samples are relatively less. Based on this, the urea-hydrolytic bacteria extracted from travertine were used to cement coral sand. The percolation test and the shear test were conducted on the cemented samples to evaluate its physical and mechanical properties. The nuclear magnetic resonance (NMR) imaging technology was adopted to quantitatively analyze the pores, and the scanning electron microscope (SEM) was applied to analyze the cementation state. Finally, the cementation effect of coral sand was comprehensively evaluated.

## Materials and methods

### Isolation and purification of bacteria

**Figure 1:**
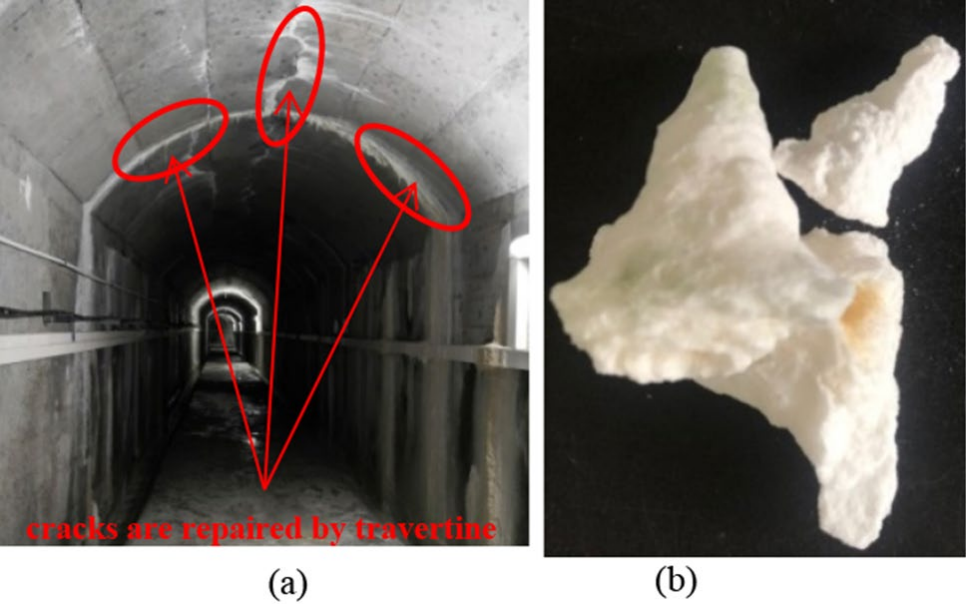
Typical travertine: (**a**) Travertine phenomenon in the tunnel; (**b**) Travertine crystals.

In the field investigation of an underground chamber of a hydropower project, the authors found that the travertine crystallization phenomenon at the seepage point in the chamber was obviously, and some of the small cracks had been repaired by travertine crystals naturally, as shown in Fig. 1a. Figure 1b is a typical travertine sample taken from this chamber. The urea-hydrolytic bacteria used in this paper were extracted from this sample.

The detailed extraction and purification of the urea-hydrolytic bacteria process was as follows:

1. 1 g of travertine sample was put into a ceramic mortar and gently crushed into powder, mixed the power with 99 mL sterile water and then poured into a 250 mL conical flask. The conical flask was shaked for 5 min to evenly disperse the powder. Thus, a diluent with a concentration of 1/100 (10^–2^) g/mL was obtained.
2. 1 mL diluent was put into a test tube containing 9 mL sterile water, and then diluted in turn to obtain 10^–3^, 10^–4^, 10^–5^, 10^–6^, 10^–7^ g/mL, respectively. The pipette gun was used to absorb 0.2 mL diluent with 10^–5^, 10^–6^, 10^–7^ g/mL, respectively, then inoculated it on the agar plate medium. A sterile coated glass rod was used to evenly dispersed it, and the coated plate medium was inverted and put into the incubator with temperature of 30 °C for 24 h.
3. When the bacteria grew out on the plate medium, used the inoculation ring to pick up the scattered and smooth colonies, and inoculated them on the agar slant medium with ‘Z’ line. After inoculation, placed the slant medium in the incubator with the same culture temperature and time.
4. When the bacteria grew out on the slant culture medium, inoculated the colonies into the plate culture medium again, and repeated for 4 ~ 5 times to obtain the purified bacterial strain.

After the bacteria was purified, 100 mL of the prepared liquid culture medium was took and put into a conical flask with a capacity of 250 mL, used the inoculation ring to inoculate the colonies in the slant culture medium into the conical flask, and then put the conical flask into a shaker with a temperature of 30 °C and a rotating speed of 120 r/min for 48 h to obtain the expanded culture liquid.

**Figure 2:**
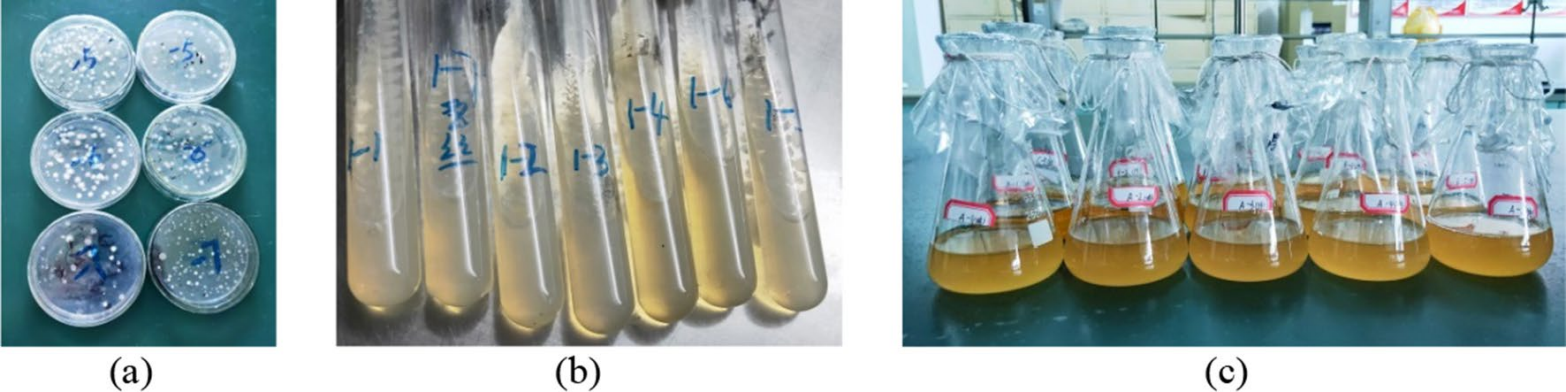
Different types of mediums: (**a**) Agar plate culture medium; (**b**) Agar slant culture medium; (**c**) Liquid culture medium.

**Figure 3:**
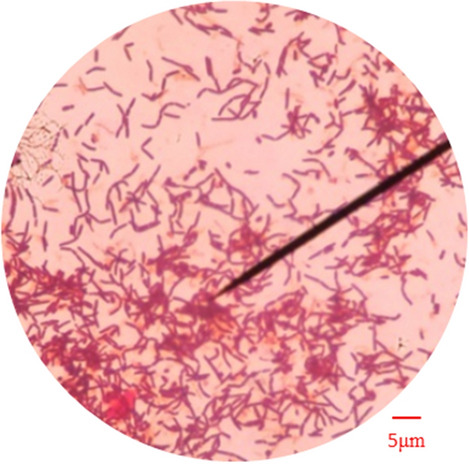
Morphology of the purified bacteria.

The bacterial concentration and urease activity were used as two indicators to measure the MICP performance of *Bacillus thuringiensis*. A wavelength of 600 nm (OD_600_) was used in the measurement, so the value of OD_600_ could represent the concentration of the bacterial solution^22^. Urease activity was measured by electrical conductivity followed the following steps: 1 mL bacterial solution was added to 9 mL 1.1 mol/L urea solution, and the change of electrical conductivity (ms/cm min) in 5 min was measured by multiplying the dilution multiple to obtain urease activity (mmol/L min)^23^.The bacterial concentration (OD_600_ value) was measured to be 1.28, and the urease activity was 13.63 mmol/L min. Under the same culture conditions, the bacterial concentration and urease activity of *Sporosarcina pasteurii* were 0.84 and 10.49 mmol/L min, respectively. Compared with *Sporosarcina pasteurii*, the bacterial concentration and urease activity of the strain isolated from the travertine sample was increased by 34.38% and 29.93%, indicating that the strain has a higher urease-producing ability. Agar plate culture medium, agar slant culture medium and liquid culture medium are shown in Fig. 2. The morphology of the purified bacteria was observed by an optical microscope as shown in Fig. 3. The medium was composed of the following components: 10 g/L peptone, 3 g/L beef extract, 5 g/L sodium chloride, and 20.02 g/L urea. To isolate the bacteria, 15 g/L agar was added to the solid culture medium (plate culture medium and slant culture medium) at the same time. Eventually, the purified bacteria was identified by 16S rDNA sequencing as *Bacillus thuringiensis*.

### Orthogonal test of bacteria culture conditions

In order to further improve the bacterial concentration and urease activity, according to relevant researches^22,24^, the urea content (20.02 g/L, 40.04 g/L, 60.06 g/L), microbial inoculation (10 mL/L, 15 mL/L, 20 mL/L), shaker speed (120 r/min, 150 r/min and 180 r/min) and incubation time (24 h, 48 h, 72 h) were slected as four influence factors to obtain the best culture conditions of *Bacillus thuringiensis*. The concentration of bacterial solution was tested by orthogonal experiment for the above four factors, and L_9_ (3^4^) orthogonal experiment was used and the orthogonal test table was shown in Table 1. The absorbance value (OD_600_) measured by spectrophotometer was taken as the concentration of bacterial solution. The detailed results are shown in Table 2.

**Table 1:** Orthogonal test table.

Level	Factor			
	Urea content (g/L)	Microbial inoculation (mL/L)	Shaker speed (r/min)	Incubation time (h)
1	20.02	10	120	24
2	40.04	15	150	48
3	60.06	20	180	72

**Table 2:** Orthogonal test results.

Test no.	Urea content (g/L)	Microbial inoculation (mL/L)	Shaker speed (r/min)	Incubation time (h)	OD_600_ value				Urease activity (mmol/L min)			
					1	2	3	Mean value	1	2	3	Mean value
1	20.02	10	120	24	1.45	1.48	1.50	1.48	13.35	13.22	13.3	13.29
2	20.02	15	150	48	1.52	1.53	1.51	1.52	13.76	13.83	13.69	13.76
3	20.02	20	180	72	1.78	1.77	1.70	1.75	14.63	14.75	14.7	14.69
4	40.04	10	150	72	1.47	1.42	1.45	1.45	11.88	11.79	11.86	11.84
5	40.04	15	180	24	1.53	1.58	1.55	1.55	13.99	14.03	13.86	13.96
6	40.04	20	120	48	1.39	1.45	1.40	1.41	11.85	11.62	11.78	11.75
7	60.06	10	180	48	1.81	1.86	1.83	1.83	15.79	15.84	15.95	15.86
8	60.06	15	120	72	1.25	1.20	1.29	1.25	11.17	11.41	11.3	11.29
9	60.06	20	150	24	1.47	1.46	1.44	1.46	11.98	12.16	12.08	12.07

The OD_600_ mean values were fell within the range from 1.25 to 1.83 and the urease activity mean values were ranging from 11.29 to 15.86 mmol/L min from Table 2. Test NO.3 and test NO. 7 had relatively higher OD_600_ values, but compared with test NO.3, test NO.7 needed less microbial inoculation and shorter culture time to achieve a higher bacterial concentration, so test NO.7 was choosed as the optimal culture condition in this paper. That was, the culture condition for *Bacillus thuringiensis* was as follows: urea content 60.06 g/L, microbial inoculation 10 mL/L, the shaker speed 180 r/min, and the incubation time 48 h. All the *Bacillus thuringiensis* used in the subsequent cement test were cultured in this condition.

### Cementation test materials

**Figure 4:**
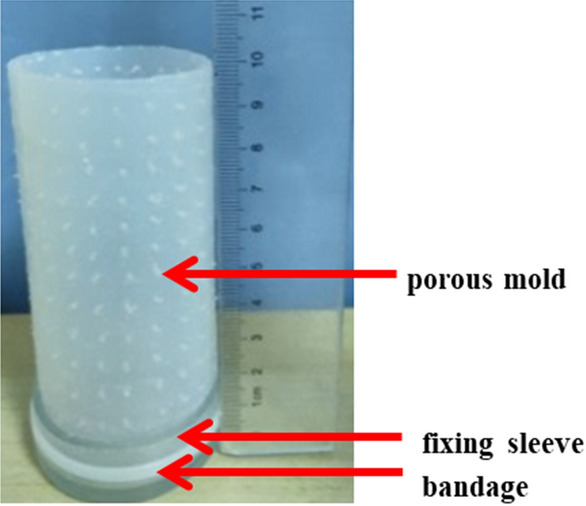
Elastic porous silicone mold.

The cementation test of coral sand was carried out by free infiltration. The molds for holding the coral sand were elastic porous silicone mold which is 50 mm in diameter and 100 mm in height. The sidewall of the mold was porous. In order to prevent the coral sand from leaking out at the bottom, a fixing sleeve with an inner diameter of 50 mm was settled at the bottom of the mold (there was a small hole in the center of the bottom of the fixing sleeve in order to facilitate the outflow of waste liquid), a bandage was utilized to bind the fixing sleeve to the bottom of the mold, as shown in Fig. 4.

**Figure 5:**
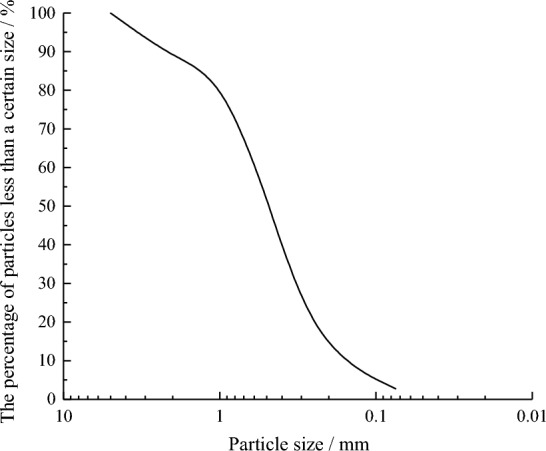
Particle distribution curve.

The specific density of the coral sand was 2.80. In order to optimize the reinforcement effect and obtain the cemented sand column with uniform strength, The samples were prepared after screening by the geotechnical standard sieve. The particle distribution curve is illustrated in Fig. 5.

### Coral sand cementation process

To study the effect of the different cementation times on the coral sand, two different cementation groups were considered, one was cemented for 7 times, the other was 14 times. In addition, the temperature and humidity in the laboratory were 25 °C and 40%, respectively.

**Figure 6:**
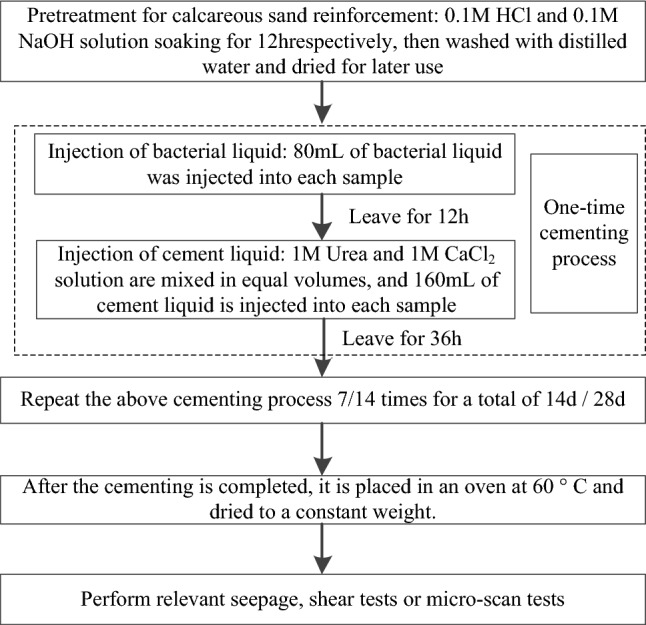
Cementation process of the coral sand.

According to Fig. 6, the cementation test was conducted by the following steps.

1. Before cementation, the coral sand was pretreated by soaked in 0.1 mol/L HCl solution and then in 0.1 mol/L NaOH solution for 12 h, respectively ^25^, and then washed with distilled water and dried to constant weight for later use.
2. The coral sand was divided into three layers to put into an elastic porous silicone mold, each layer was slightly vibrated and compacted, roughened the surface of each layer to compact the layers. Then the distilled water was injected from the top of the mold to remove the air between the sand particles.
3. 80 mL bacteria solution was injected into the sand samples and the bacteria stayed in the columns for 12 h until they were fully attached to the sand particles.
4. The cementing solution was a mixture of 1 mol/L calcium chloride and 1 mol/L urea by the same volume. After they were well mixed, the cementing solution (160 mL 0.5 mol/L) was injected into the sand sample and allowed to stand for 36 h until the bacteria can fully hydrolyzed the urea and combined the calcium ions in the solution to produce calcium carbonate.
5. Steps (3) and (4) are collectively referred to as one cementation time, and they were repeated for 7 or 14 times to cement the coral sand.
6. After reaching the cementation times of each group, the cemented samples were put into the thermostat at 60 °C until they were reached the constant weight, and the follow-up related macroscopic physical mechanics or microscopic tests were carried on them.

**Figure 7:**
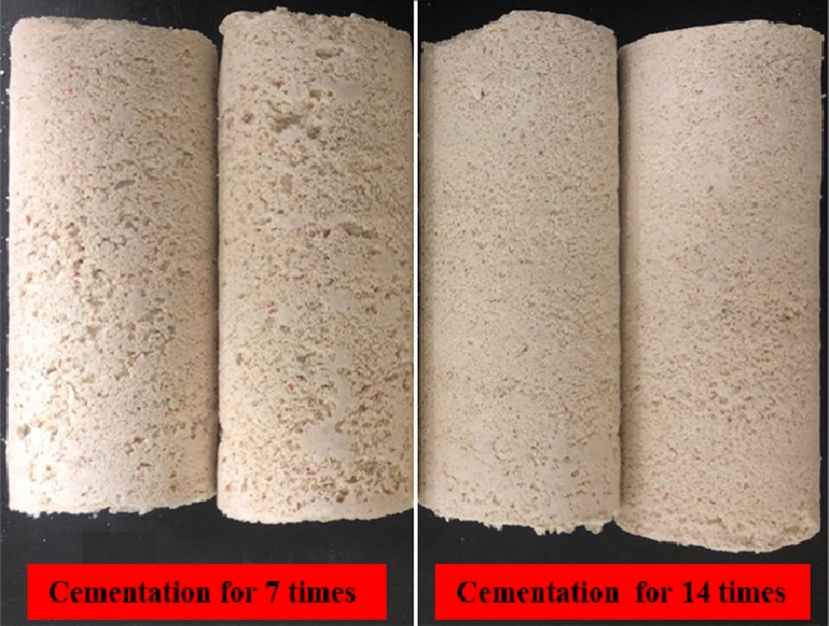
Typical microbial cemented coral sand samples under different cementation times.

Typical cemented sand samples were shown in Fig.7.

## Results and discussion

### Calcium carbonate content

Relevant studies^23^ have shown that when the content of calcium carbonate is higher than 60 kg/m^3^, the strength of the sand sample will be significantly improved, so calcium carbonate content is one of the important factor to measure the reinforcement effect. Calcium carbonate deposits in cemented siliceous sand samples is usually measured by the pickling process^26^, but coral sand has a high content of calcium carbonate, the pickling process is not suitable. Therefore, the weight difference between the coral sand sample before and after cementation is measured to distinguish the calcium carbonate precipitation in this paper.

By weighing the mass of the different group samples before and after cementation under dry conditions, the calcium carbonate mass generated in the sand column was calculated, and its content in the samples are calculated through conversion.

**Figure 8:**
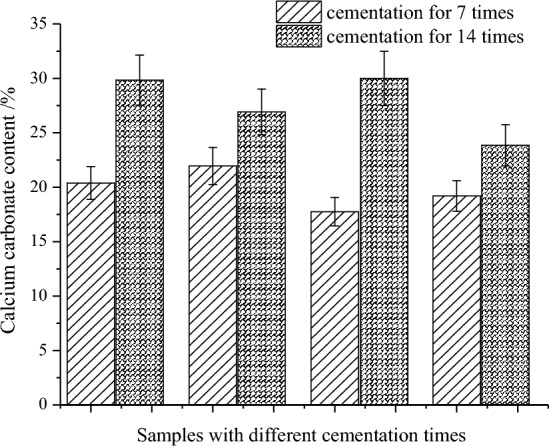
Calcium carbonate content in the sand samples under different cementation times.

Figure 8 shows that the calcium carbonate content is between 17.75 and 21.95% in the smples under 7 cementation times, while it is between 23.85 and 30.00% under 14 times, with mean values of 19.82% and 27.66%, respectively. In comparison, the mean content for the samples of 14 cementation times is about 8.00% higher than that of 7 times, indicating that the more cementation cycles, the more calcium carbonate precipitation in sand samples. It can also be seen from the typical sample in Fig. 7 that the surface of the sample under 14 cementation times is obviously smoother and has fewer pores than 7 times, indicating that more calcium carbonate is generated to fill the pores between and inside the sand particles.

The calcium carbonate content in the sand sample after 7 cementation times by Peng et al.^21^ is 10.70%. The content in the in-situ grouting and reinforcement test by Leon et al.^27^ ranges from 12.60 to 27.30% at different measuring points. The precipitation content by Zhao et al.^28^ is characterized by the washing the samples in HCl solution (0.1 M) to dissolve precipitated carbonate, and the maximum calcium carbonate content is about 14.40%. Compared to these researches, the *Bacillus thuringiensis* produced a higher calcium carbonate content in the coral sand samples in this study, which indicating that the *Bacillus thuringiensis* has a good ability to generate calcium carbonate.

### Permeability resistance

The seepage characteristic is equally an important factor influencing the mechanical strength of cemented bodies while evaluating the reinforcement effect. The HYS-4 rock percolation meter was used to conduct percolation tests on the sand samples under different osmotic pressures (0.5 MPa, 1.0 MPa, 1.5 MPa, 2.0 MPa, 2.5 MPa) and different lateral stresses (1.0 MPa, 1.5 MPa, 2.0 MPa, 2.5 MPa, 3.0 MPa) to study the percolation characteristics. Before the test, the sample was vacuumed and saturated with distilled water for 24 h to ensure that the seepage flow in the sample was single-phase.

According to Formula (1) ^29^, the permeability coefficient of samples under different lateral stresses and osmotic pressure can be calculated.

1 $${\text{k}} = \frac{{{\text{Q}}L\gamma_{{\text{w}}} }}{{\Delta {\text{PA}}}}$$

where, *k* is the permeability coefficient of the sand sample, *Q* is the flow rate of fluid through the sand sample in unit time, *L* is the length of the sand sample, *γ*_*w*_ is the water intensity, *ΔP* is the head difference at both ends of the sand sample, *A* is the cross-sectional area of the sand sample.

**Figure 9:**
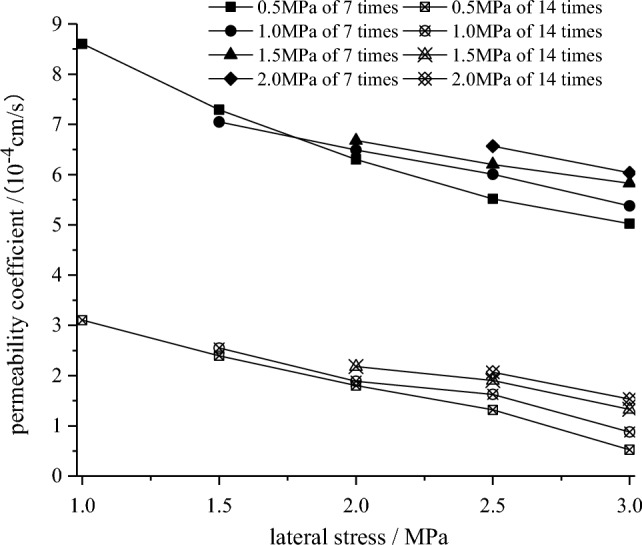
Permeability coefficient of the sand samples under different osmotic pressure with different cementation times.

Preliminary tests show that the permeability coefficient of calcareous sand sample before cemented is about 10^−2^ cm/s, after cemented by different cycles, the authors can observe from Fig. 9 that the permeability coefficient of the samples are down to 10^–4^ cm/s, falling by two orders of magnitudes, this is consistent with previous researches^30,31^,which illustrating that the *Bacillus thuringiensis* can also fill and cement coral sand particles well, and improve the permeability of the solidified body. In comparison, the permeability coefficient of the sample under 14 cementation times is more than 60% lower than that under 7 times. That is, *Bacillus thuringiensis* can induce more calcium carbonate precipitation with more cementation cycles. The samples are filled with the precipitated CaCO_3_ and the adjacent sand particles are cemented by them, the internal pores of the sample decrease, thus reducing the permeability coefficient.

### Shear strength

**Figure 10:**
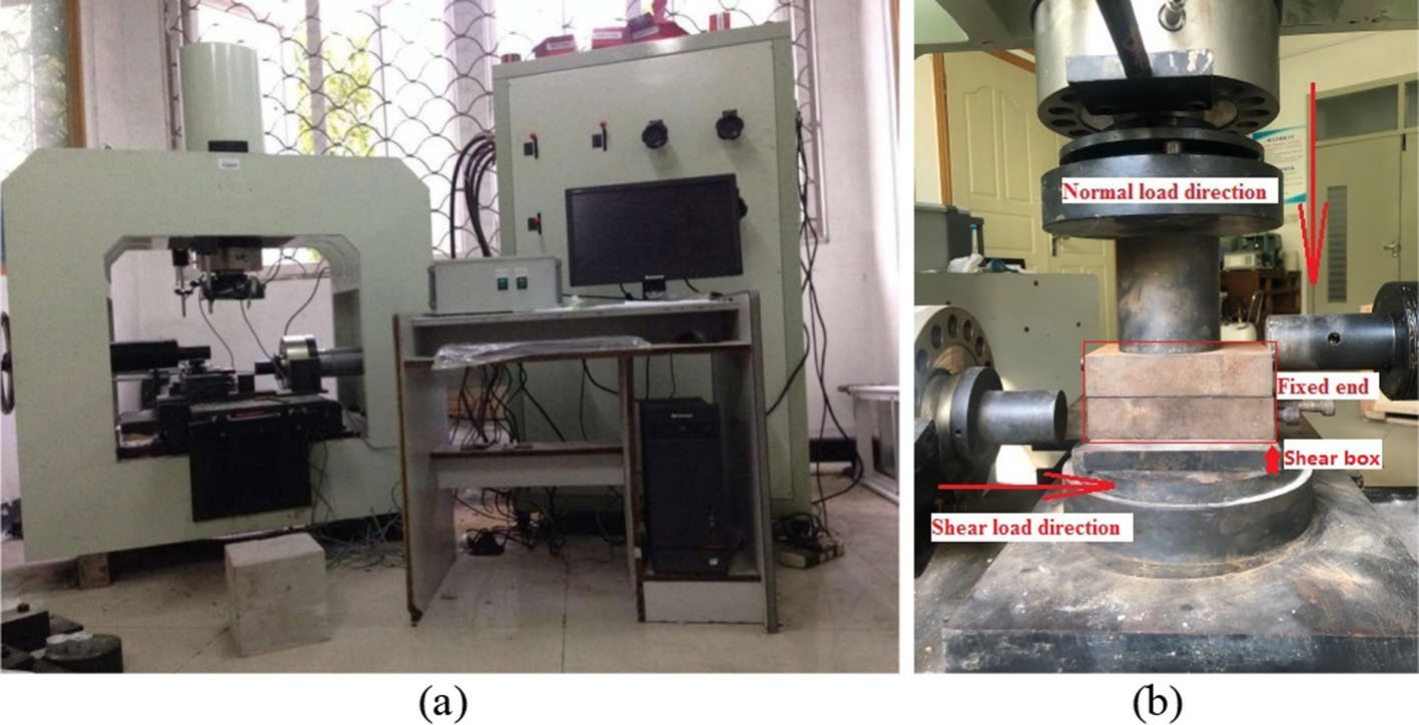
Direct shear apparatus: (**a**) YZW1000 computerized direct shear testing device; (**b**) Shear loading device.

In order to analyze the shear properties, the shear test is conducted on the sand columns. The shear tests were conducted by using direct shear apparatus (Fig. 10a). The shear loading device is shown in Fig. 10b. Four normal stresses of 400 kPa, 600 kPa, 800 kPa, and 1000 kPa are considered for these test. The shearing rate was 1%/min^32^ and the shearing stop condition was when the shear strength was maintained at a relatively stable value.

**Figure 11:**
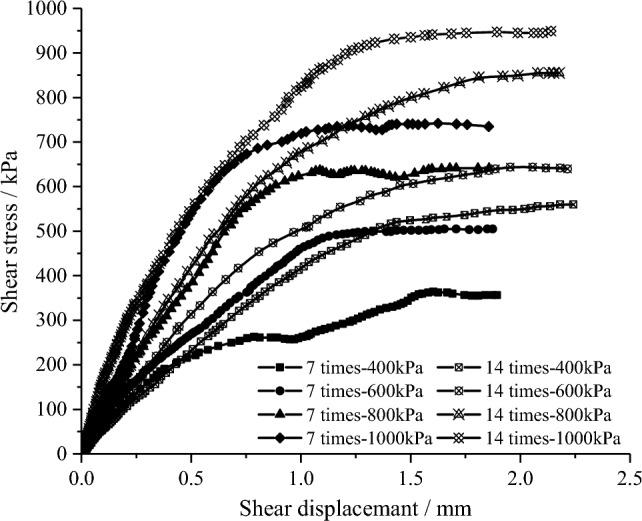
Shear stress–shear displacement curve of the sand samples under different cementation times.

The shear stress–shear displacement curves of typical samples are shown in Fig. 11. Under different cementation times, the shear stress–shear displacement curves under shear load are basically the same, that is, the curve gradually tends to be stable after reaching the peak strength and presents the state of shear flow. The difference is that with the increase of the cementation time, the shear strength under the same normal stress increases to different degrees. Compared with 7 cementation times, the shear strength of the samples under 14 times increases by 41.2%, 36.5%, 32.3% and 29.6%, respectively. At the same time, the corresponding shear displacement increases when the curve reaches the relative stable peak strength.

**Figure 12:**
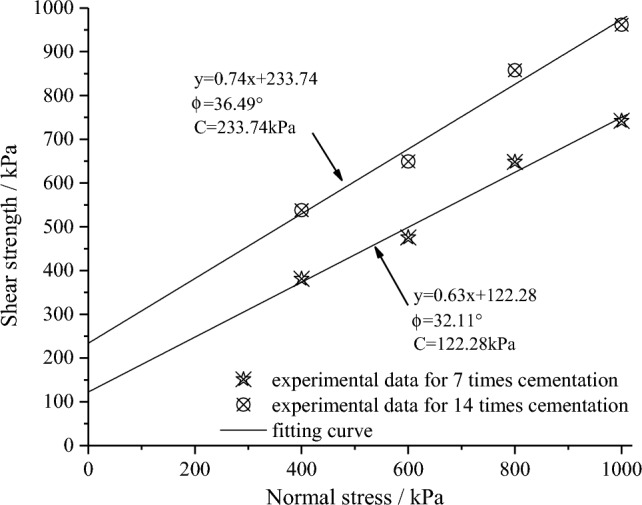
Shear strength parameters of cemented samples under different cementation times.

To further analyze the influence of the two different cementation times on the shear strength parameters, linear fitting was performed for the shear strength under different normal stresses, and corresponding shear strength parameters were obtained, as shown in Fig. 12.

The internal friction angle and cohesion of the sand samples for different cementation times are 32.11° and 36.49°, 122.28 kPa, and 233.74 kPa, respectively. Compared with 7 times, the angle of internal friction and the cohesion of 14 times is improved by 13.63% and 91.15%. It shows that with the increase of the cementation times, the cohesion is greatly improved, which is consistent with the conclusion by Wu et al.^33^, and under the same cementation cycles, the cohesion and the internal friction angle in this paper are over 1.5 times and 1.1 times bigger than the results.

### Nuclear magnetic resonance analysis

Nuclear magnetic resonance (NMR) was used to detect the hydrogen atoms inside the porous media in a low-intensity magnetic field, to obtain the T_2_ distribution spectrum of the fluid in the pores of the material, and thereby to analyze the microscopic pore structure characteristics inside the porous media^34^. MacroMR12-110H-1 NMR imaging analysis system is used to scan the samples. According to the T_2_ distribution spectrum and relevant characteristic parameters, the structural characteristics of microscopic pores inside the sand samples are quantitatively analyzed.

**Figure 13:**
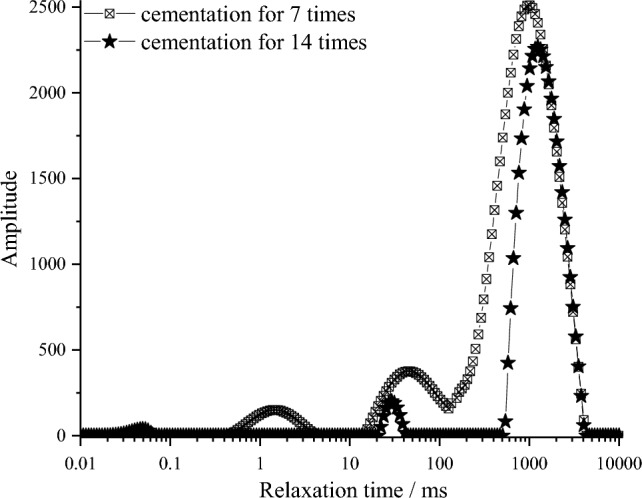
Typical sample T_2_ spectrum distribution under different cementation times.

The T_2_ spectrums of a typical saturated cemented sample are shown in Fig. 13. The abscissa represents the relaxation time and the ordinate represents the amplitude. The longer the relaxation time, the larger the pores are. The larger the amplitude, the more the pores are. The T_2_ spectrums of the samples have multiple peaks, and the main peak is around 1000 ms. The overall distribution pattern of the atlas is rightward. It is mainly dominated by long relaxation time and slow relaxation speed, indicating that the small pores in the samples are well sealed.

The NMR spectral area can quantitatively describe the changes in the number and size of the pore structure of the sample^35^. According to the imaging principle, the proportion of fluid filling in different pores is used to reflect the pore distribution, the pores are divided into small pore (< 10 ms), medium pore (10 ~ 100 ms), and large pore (> 100 ms) based on the transverse relaxation time of the T_2_ spectral area. Then the T_2_ spectral area distribution under each pore size is calculated.

Table 3 shows the change of pore area of sample T_2_ spectrum under different cementation time. In general, macropores provide most of the pore area^36^. It can be seen from Fig. 13 that with the increase of the cementation times, the amount of calcium carbonate generated by the amplitude of T_2_ spectrum increases, which can better fill the pores between and inside the sand particles. Therefore, the number of pores under each pore size decreased significantly, and the total pore area decreased by 43.67%.

**Table 3:** Different pore sizes area distribution of T_2_ spectrum in the coral sand sample.

Cementation time	Classification	Pore categories			Total pore area
		Small pores	Medium pores	Big pores	
7	T_2_ spectrum area	2880.47	7451.99	67,360.28	77,692.74
	Proportion/%	3.71	9.59	86.70	100.00
14	T_2_ spectrum area	314.05	1274.26	42,179.68	43,767.99
	Proportion/%	0.72	2.91	96.37	100.00
The area of T_2_ spectrum decreases proportionally/%		89.10	82.90	37.38	43.67

**Figure 14:**
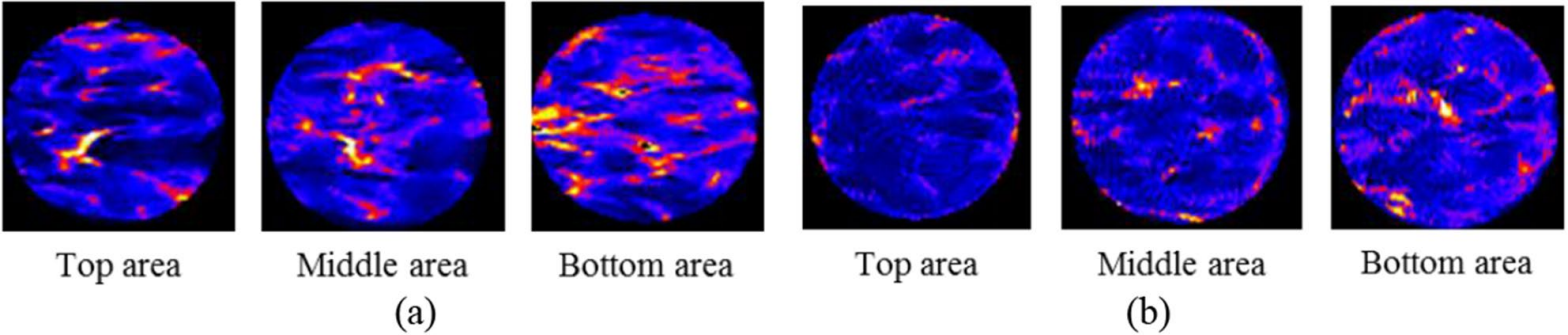
NMR cross section scanning of the sand samples under different cementation times: (**a**) 7 times; (**b**) 14 times.

In order to further observe the sample’s filling and cementing effect along with the height, the top, middle, and bottom sections were scanned and analyzed. Figure 14 shows the scanning images of the two samples, whereby the black is the background, blue is the coral sand and calcium carbonate cementation, and red is the region where the water molecules are located. The brighter the red, the denser the water molecules and the more pores there are.

From the analysis of the scanning section, the pores of 14 times is less than 7 times, illustrating that the whole filling effect of 14 times is better than 7 times. Because in the cementation process, the cementation cycles of 7 times is relatively less, the quantity of the calcium carbonate precipitation is little, due to insufficient accumulation of calcium carbonate, the filling effect of large size pores is poor, the scan results have much red highlighted area, this is consistent with the T_2_ spectrum distribution areas. By comparing the cross-section profiles of the two samples, it is found that the filling effect of the samples is weakened from top to bottom. With the reinforcement experiment going on, the generated calcium carbonate can well fill the pores between the particles and cement the adjacent particles. In the process of calcium carbonate precipitation, the pores are populated gradually from top to bottom of the sample, the infiltration channels of the bacterial and the cementing solution in the subsequent cementing process are reduced. Thus the precipitated calcium carbonate is weakened, so the CaCO_3_ crystals are homogeneous distributed along in the samples, the red part is getting more and more prominent from the top area to the bottom area of the sample, means more pores in the samples.

### Analysis of SEM microstructure characteristic

**Figure 15:**
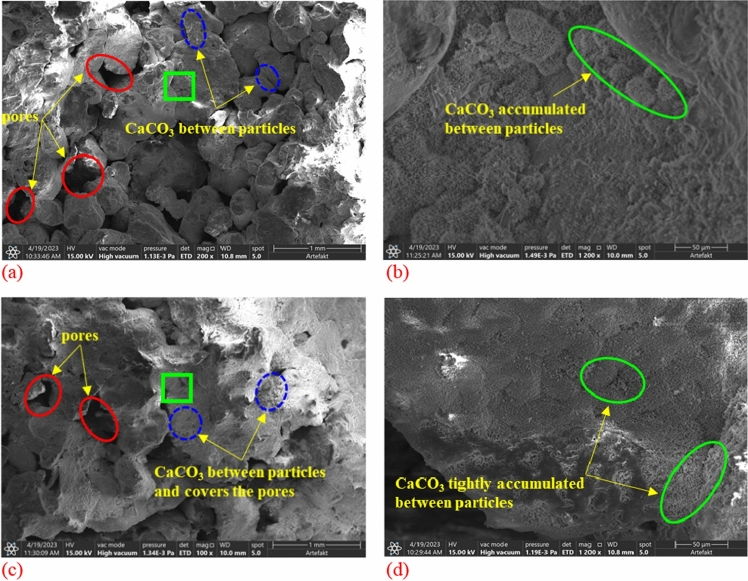
The microstructure of calcium carbonate precipitation in the sand samples under different cementation times: (**a**) magnify 200 × under 7 times; (**b**) magnify 1200 × under 7 times; (**c**) magnify 200 × under 14 times; (**d**) magnify 1200 × under 14 times.

SEM Scanning of broken samples after shear failure under different cementation times, the cementation statuses of coral sand particles are observed at 200 and 1200 times magnification respectively, as shown in Fig. 15. The results show that the calcium carbonate produced by microbial mineralization is mainly divided into two states. Firstly, the CaCO_3_ crystals are between coral sand particles. Secondly, CaCO_3_ crystals cement two adjacent coral sand particles. Under the MICP effect, a large amount of scattered calcium carbonate precipitation centered on the nucleus of the microorganism is produced, with the increase of precipitation and accumulation, the pores between the sand particles are slowly filled, maked the two sand particle cemented with each other. Through comparison, it can be seen that compared with 7 cementation times, there is a certain degree of reduction in the pores between particles, and the generated calcium carbonate precipitation more tightly between particles and on the surface of particles after 14 cementation times.

**Figure 16:**
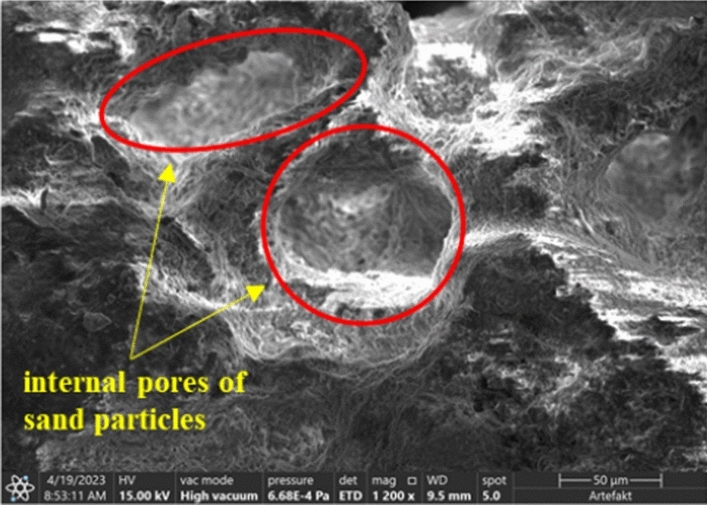
Calcareous sand particles before cementation treatment.

At the same time, SEM scanning images of the calcareous sand particles before cementation treatment were added as a reference, as shown in Fig. 16. It can be seen that calcareous sand particles also contain internal pores, the calcium carbonate precipitation not only cemented the calcareous sand particles but also filled the pores inside the particles.

The SEM images of the samples under 7 cementation times show that there are more calcium carbonate deposits between the loose grains, but less calcium carbonate is used to cement two adjacent sand particles. The less effective contact between particles results in more pores between sand particles and less compact cementation. From a macro perspective, it shows that the permeability coefficient is large and the shear strength is not high.

The sample under 14 cementation times has less pores and more calcium carbonate between sand particles than 7 times. The precipitated calcium carbonate increases, the uncemented portion between particles decreases, resulting in that more calcium carbonate is generated around the sand particles, the size of the calcium carbonate increases with the increase of the cementation cycles. On the one hand, sand particles can be better coated and filled with pores between sand particles, on the other hand, the adjacent uncemented coral sand particles are more easily cemented by the larger calcium carbonate precipitation, which makes the two adjacent sand particles tightly cemented into a whole. Macroscopically, the permeability and shear strength of the cemented samples are further improved.

### Evolution model under different cementation times

**Figure 17:**
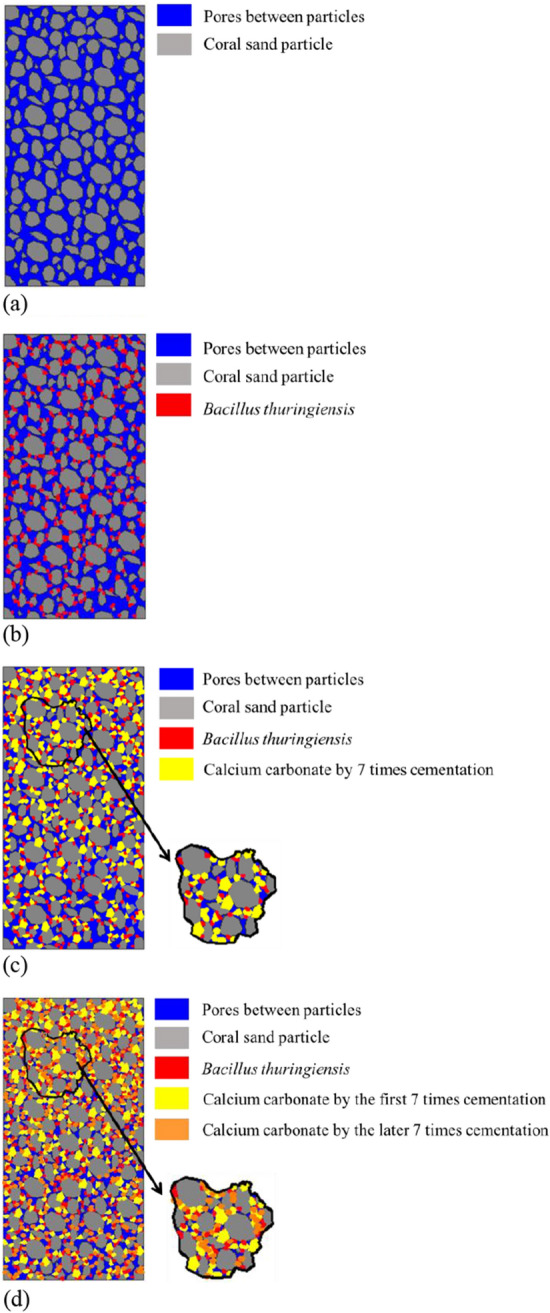
Schematic diagram of MICP-cemented sand sample evolution model: (**a**) Pore distribution of the sand sample before reinforcement; (**b**) *Bacillus thuringiensis* adsorbed on the surface of sand particles; (**c**) Calcium carbonate distribution in the sand sample for 7 cementation times; (**d**) Calcium carbonate distribution in the sand sample for 14 cementation times.

Based on the above analysis, the filling effect and cementation state between coral sand particles can be represented by the evolution model, as shown in Fig. 17.

It can be seen from Fig. 16a–d that different sizes of coral sand naturally forms the pores of varying sizes in the porous mold. And the loose sand particles are enclosed by negatively charged bacteria in the cell walls as the bacteria solution is injected. Due to the metabolism of the bacteria, the produced carbon dioxide dissolves in water to form carbonic acid root, as the cement solution is injected, the calcium ions in the cementing solution accumulate around the bacteria and bind to the carbonate in the solution. Thus to form a calcium carbonate precipitation with bacteria as a nucleation site, the precipitation has a cementing effect and can fill the pores between coral sand particles, as well as cement adjacent sand particles.

On the one hand, when the coral sand is cemented by the infiltration method, during the injecting process of the bacterial and cementing solution, the generated calcium carbonate will gradually cement and fill the pores on the upper part of the sample, causing the infiltration channel of the solution to be blocked at the later stage, as a result, the sample shows the phenomenon of inhomogeneity from top to bottom.

On the other hand, due to the existence of various graded sand particles in the sample, the pore size distribution in the sample is relatively uniform. When the cementation cycle is less, the amount of precipitated calcium carbonate is less and the size is not large, so the filling effect of the large and medium pores is not good. With the increase of cementation times, the calcium carbonate precipitation increase and cement with each other, thus increasing the aggregate size of calcium carbonate, so the filling effect of large, medium and small pores are evidently improved. It can be seen from microcosmic test results that the number of pores in 7 times is significantly reduced compared with 14 times, and the cementation between sand particles are more closely and tightly. From the perspective of macro-physical and mechanical properties, the permeability decreases and the strength increases.

## Conclusions

This paper analyzes the formation process and the microbial environment of travertine, a new type of urea-hydrolytic bacteria named *Bacillus thuringiensis* is extracted from the travertine. The relatively better culture conditions are selected after purification and performing orthogonal tests on the bacteria. Then two groups of the coral sand cementation tests are conducted with *Bacillus thuringiensis*. The following conclusions can be drawn.

1. The optimal culture conditions for *Bacillus thuringiensis* are as follows: 60.06 g/L urea, 10 mL/L bacterial inoculation, 180 r/min shaking speed of the shaker, and 48 h culture time.
2. The permeability coefficient of the coral sand samples are as low as 10^−4^ cm/s. Generally, the more cementation times, the smaller the permeability coefficient and the greater the shear strength. Compared with the sand samples with commonly used *Sporosarcina pasterurii*, the cohesive force and the internal friction angle of the sand samples cemented by *Bacillus thuringiensis* is increased by more than 1.5 times and 1.1 times respectively.
3. NMR results show that the distribution of calcium carbonate along the height of the sand sample is uneven, because the calcium carbonate generated in the cementing process will block the pores between sand particles, resulting in the subsequent infiltration channels of bacterial and cementing solution reduce, thereby affecting the formation of calcium carbonate and downward sedimentation effect, further leading to the porosity of the sample become bigger and more from top to bottom.
4. The SEM analysis results and the evolution model of the samples under different cementation times show that the generated calcium carbonate could effectively fill the pores between the sand particles and cement the adjacent sand particles. The more cementation times, the more calcium carbonate is produced and the more significant effect of effective filling and cementing of sand particles, especially for the small and medium pores.

In the follow-up research, we will consider other factors that affect the cementing effect, establish a correlation between certain influencing factors and mechanical strength, focus on the static and dynamic properties of the solidified body, optimize the process of microbial reinforcement of coral sand to reduce the cementation time and improve the cementation performance, and apply the optimized reinforcement process to the actual project to provide corresponding management ideas for foundation consolidation and anti-seepage treatment in coral sand areas.

## References

[1] Rahimi F (2016). Study of the long-term changes in water escape at the lar dam reservoir in Northern Iran. Dam Eng..

[2] Qdais HA (2019). Leachability of heavy metals from stabilized/solidified mine tailing in Russia. J. Eng. Res..

[3] Ishihara K, Koga Y (1981). Case studies of liquefaction in the 1964 Niigata earthquake. Soils Found.

[4] Zhu, Z., Kham, M., Fernandes, V. A. & Lopez Caballero, F. Dynamic response of a central clay core dam under two-component seismic loading. In* The International Conference on Embankment Dams, Springer, Cham*, 231–236 (2020).

[5] Zhang XC (2021). Characteristics and prevention mechanisms of artificial slope instability in the Chinese Loess Plateau. CATENA.

[6] Chu J, Stabnikov V, Ivanov V (2012). Microbially induced calcium carbonate precipitation on surface or in the bulk of soil. Geomicrobiol. J..

[7] Qing CX, Pan F, Wang RX (2010). Cementation of sand grains based on carbonate precipitation induced by microorganism. Sci. China Technol. Sci..

[8] Khan Md NH, Amarakoon G, Shimazaki S, Kawasaki S (2015). Coral sand solidification test based on microbially induced carbonate precipitation using ureolytic bacteria. Mater. Trans..

[9] Ford TD, Pedley HM (1996). A review of tufa and travertine deposits of the world. Earth Sci. Rev..

[10] Fouke BW (2011). Hot-spring systems geobiology: Abiotic and biotic influences on travertine formation at Mammoth Hot Springs, Yellowstone National Park, USA. Sedimentology.

[11] Sugihara C, Katsunori Y, Tomoyo O, Chizuru T (2016). Transition of microbiological and sedimentological features associated with the geochemical gradient in a travertine mound in northern Sumatra, Indonesia. Sediment Geol..

[12] Javad K, Mohsenm R, Mohammad AA (2018). Isolation and identification of halophilic and halotolerant bacteria from badabe-surt travertine spring, Kiasar, Iran, and investigation of calcite biomineralization induction. Geomicrobiol. J..

[13] Riding R (2000). Microbial carbonates: The geological record of calcified bacterial-algal mats and biofilms. Sedimentology.

[14] Dupraz C, Reid RP, Braissant O, Decho AW (2009). Processes of carbonate precipitation in modern microbial mats. Earth Sci. Rev..

[15] Okyay TO, Nguyen HN, Castro SL, Rodrigues DF (2016). CO_2_ sequestration by ureolytic microbial consortia through microbially-induced calcite precipitation. Sci. Total Environ..

[16] Zhang Y, Guo HX, Cheng XH (2015). Role of calcium sources in the strength and microstructure of microbial mortar. Constr. Build Mater..

[17] Karim R, Mashaallah K, Reza HS, Mohammad RN (2016). Effect of injected bacterial suspension volume and relative density on carbonate precipitation resulting from microbial treatment. Ecol. Eng..

[18] Rong H, Qian CX, Li LZ (2012). Influence of molding process on mechanical properties of sandstone cemented by microbe cement. Constr. Build Mater..

[19] Song CP, Wang CY, Derek E, Zhi S (2022). Compressive strength of MICP-treated silica sand with different particle morphologies and gradings. Geomicrobiol. J..

[20] Nafisi A, Liu QW, Montoya BM (2021). Effect of stress path on the shear response of bio-cemented sands. Acta Geotech..

[21] Liu L (2019). Strength, stiffness, and microstructure characteristics of biocemented calcareous sand. Can. Geotech. J..

[22] Teng FC, Sie YC, Ouedraogo C (2021). Strength improvement in silty clay by microbial-induced calcite precipitation. Eng. Geol. Environ..

[23] Whiffin VS, Van PLA, Harkes MP (2007). Microbial carbonate precipitation as a soil improvement technique. Geomicrobiol. J..

[24] Xiao Y, Deng HF, Li JL, Chen XZ (2022). Shear performance and reinforcement mechanism of MICP-treated single fractured sandstone. Front. Earth Sci..

[25] Sun XH, Miao LC, Tong TZ, Wang CC (2018). Effect of methods of adding urea in culture media on sand solidification tests. Chin. J. Geotech. Eng..

[26] Cheng L, Shahin MA, Ruwisch RC (2014). Bio-cementation of sandy soil using microbially induced carbonate precipitation for marine environments. Géotechnique..

[27] Leon AP (2010). Quantifying biomediated ground improvement by ureolysis: Large-scale biogrout experiment. J. Geotech. Geoenviron..

[28] Zhao Q (2014). Factors affecting improvement of engineering properties of MICP-treated soil catalyzed by bacteria and urease. J. Mater. Civil Eng.

[29] Neumann W, Breuer D, Spohn T (2016). Water-rock differentiation of icy bodies by darcy law, stokes law, and two-phase flow. Proc. Int. Astron. Union.

[30] Behzadipour H, Sadrekarimi A (2021). Biochar-assisted bio-cementation of a sand using native bacteria. Bull. Eng. Geol. Environ..

[31] Ivanov V, Chu J (2008). Application of microorganisms to geotechnical engineering for bioclogging and biocementation of soil in situ. Rev. Environ. Sci. BioTechnol..

[32] Chou CW, Seagren EA, Aydilek AH, Lai M (2011). Biocalcification of sand through ureolysis. J. Geotech. Geoenviron..

[33] Wu CC (2019). Investigation on the shear behavior of bio-cemented sand under different influencing factors. J. Civil Environ. Eng..

[34] Wang YZ (2022). Analysis of freeze-thaw damage and pore structure deterioration of mortar by low-field NMR. Constr. Build Mater..

[35] Li C (2019). Field experimental study on stability of bio-mineralization crust in the desert. Rock Soil Mech..

[36] Li C (2017). The strength and porosity properties of micp-treated aeolian sandy soil. Mech. Eng..

